# Auricular Electroacupuncture for Late Posttraumatic Epilepsy after Severe Brain Injury: A Retrospective Study

**DOI:** 10.1155/2019/5798912

**Published:** 2019-10-13

**Authors:** Cui-Cui Shen, Jin-Feng Jiang

**Affiliations:** ^1^Department of Acupuncture and Moxibustion, The Affiliated Wuxi No. 2 People's Hospital of Nanjing Medical University, Wuxi 214000, Jiangsu Province, China; ^2^School of Acupuncture and Chinese Tuina, Nanjing University of Chinese Medicine, Nanjing 210023, Jiangsu Province, China

## Abstract

**Background:**

Posttraumatic epilepsy (PTE) is a common complication of traumatic brain injury (TBI), which seriously affects patients' survival and recovery. Vagus nerve stimulation (VNS) is a nonpharmacological therapy for epilepsy. The auricular branch of the vagus nerve (ABVN) is the only peripheral branch and has antiepileptic effects, but the efficacy of ABVN stimulation as treatment of late PTE is uncertain. We retrospectively analyzed the clinical efficacy of ABVN stimulation by auricular electroacupuncture for the treatment of late PTE, and investigated the influence of sodium valproate and edaravone on the anti-PTE effects of auricular electroacupuncture.

**Method:**

Univariate and multivariate logistic regression analyses were used to investigate the relationship of age, cause of PTE, use of auricular electroacupuncture, sodium valproate, and edaravone with the incidence of late PTE. To compare the curative effects of auricular electroacupuncture, 89 cases of late PTE were divided into an auricular electroacupuncture and a control group according to whether they were treated with auricular electroacupuncture. We further analyzed the influence of sodium valproate and edaravone on the effects of the treatment of PTE with auricular electroacupuncture.

**Results:**

Among age, cause, use of auricular electroacupuncture, sodium valproate, and edaravone, the use of auricular electroacupuncture was associated with significantly reduced incidence of late PTE (*P* < 0.05). Compared with the control group, there were more seizure-free cases in the auricular electroacupuncture group (*P* < 0.01). The total effective rate of the auricular electroacupuncture group was 90%. The seizure-free rate among patients treated with auricular electroacupuncture was significantly reduced, regardless of the use of sodium valproate or edaravone (*P* < 0.05).

**Conclusion:**

Auricular electroacupuncture can reduce the incidence of late PTE and is a safe and economical therapy for late PTE.

## 1. Introduction

Traumatic brain injury (TBI) refers to brain injury caused by an external mechanical force such as a blow to the head, concussive forces, acceleration–deceleration forces, a blast injury, or a projectile missile, such as a bullet [1]. According to the Glasgow Coma Scale (GCS), the severity of TBI can be classified into 3 categories: mild TBI, ≥13; moderate TBI, 9–12; and severe TBI, ≤8. Posttraumatic epilepsy (PTE) is a common complication of TBI, which is proportional to the severity of TBI. Depending on the interval between the occurrence of TBI and the first seizure, PTE is divided into 3 types: immediate (<24 h), early (1–7 days), and late seizures (>1 week after TBI) [2]. Following an injury, the brain initiates immediate neuronal and glial responses, leading to significant cell loss and neural circuit changes, which can disrupt the balance between excitatory and inhibitory neurotransmission; moreover, inflammation and increased blood–brain barrier permeability are involved in the pathological mechanisms of PTE [3]. Although multiple antiepileptic drugs (AEDs) are used in clinical settings, approximately one-third of epileptic patients experience drug-refractory seizures and even more experience AED-related adverse effects, which compromise their quality of life [4]. In addition, prophylactic AEDs are effective only against early PTE, but not late PTE, neurological deficits, or mortality [5].

Vagus nerve stimulation (VNS) is an emerging alternative therapy for patients with epilepsy, especially for seizures refractory to drugs or surgical therapy [6]. In 1985, it was reported for the first time that stimulation of the vagus nerve had antiepileptic effects [7]. VNS was approved by the FDA for the treatment of focal epilepsy in adults and children over 12 years old. Besides refractory epilepsy, VNS is also used for depression and is being considered for the treatment of obesity and memory deficits [8]. The mechanism of action of VNS is based on the following: as the vagal afferent projects to the nucleus tractus solitarius (NTS), and the NTS diffuses to other brain structures where the epileptic nidus is located, desynchronization is observed on the electroencephalogram (EEG) [9]. Recently, a clinical experiment indicated that activation of the locus coeruleus (LC)-related noradrenergic (NE) system is associated with the antiepileptic effect of VNS [10]. Another study demonstrated improved efficacy of VNS therapy for seizure control over time, as revealed by the increasing mean reduction in seizure frequency [11]. Because of the stimulation parameters, some notable side effects of VNS include cough, vocal alteration, hoarseness, and tingling [12]. Therefore, an alternative method for utilizing the mechanism of VNS is required.

Auricular point is a component of traditional Chinese medicine, attributed to traditional auricular point. In contrast, the modern auricular point derives its origin from Paul Nogier's discovery [13]. Anatomically, the auricular branch of the vagus nerve (ABVN) is the only peripheral branch, and mainly concentrates on the cymba conchae and cavum conchae of the external ear [14]. Many animal and clinical experiments have indicated that auricular vagus nerve stimulation can suppress epileptiform activity and reduce seizure frequency, via the “auriculovagal afferent pathway,” in which the NTS plays an important role [15–19]. Laboratory findings show that VNS via auricular electroacupuncture exerts a combination of anti-inflammatory and neurotrophic actions through the NTS in epileptic models [20].

In this study, we retrospectively analyzed the effects of auricular electroacupuncture on the incidence of late PTE and assessed the influence of sodium valproate and edaravone on the observed relationship.

## 2. Methods

### 2.1. Ethics and Study Population

**Figure 1:**
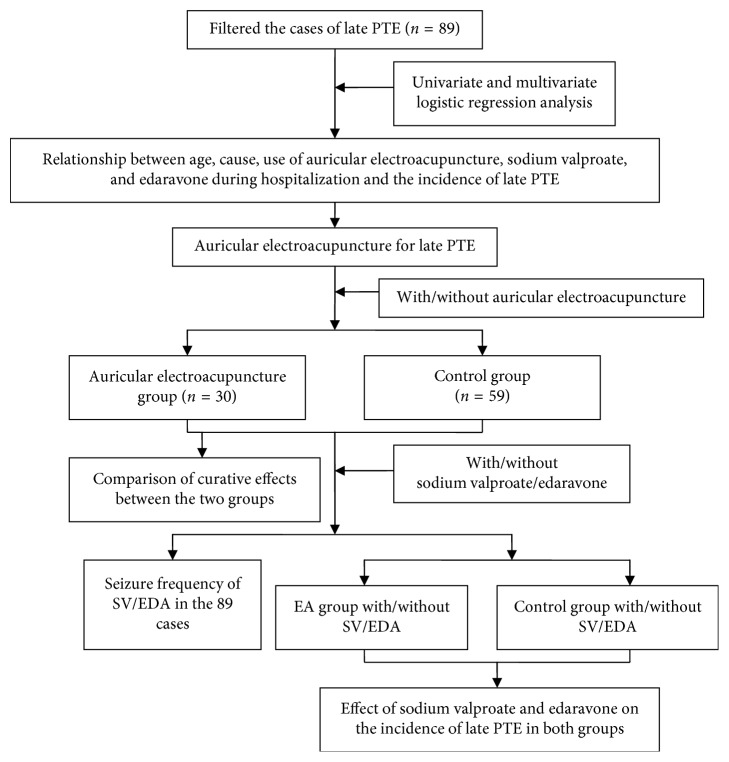
Flow diagram of the study.

This study was conducted in accordance with the principle of the Declaration of Helsinki, and the study protocol was approved by the Ethics Committee of Jiangsu Provincial Second Chinese Medicine Hospital, the Second Affiliated Hospital of Nanjing University of Chinese Medicine (No. NJDEZYYZJ201602). Because of the retrospective nature of the study, the requirement of patient consent for inclusion was waived. All information was obtained from the medical records department of Nanjing Zijin Hospital. Cases from January 2012 to December 2013 were included. The frequency of late PTE was determined based on the number of times diazepam was prescribed. The raw data from each case were recorded in Microsoft Excel (Microsoft Corp., Redmond, WA), including each patient's name, gender, age, pathogenesis, location, seizure frequency, and treatment. The details of the study design are presented in Figure 1.

### 2.2. Inclusion Criteria for Late PTE

Patients were diagnosed with severe TBI based on the following inclusion criteria: (a) comatose state for over 6 hours after injury or increasing loss of consciousness level; (b) signs of central nervous system involvement; (c) obvious changes in vital signs; and (d) GCS ≤8. Furthermore, cases that met the following inclusion criteria for late PTE were included in the study:

1. History of TBI
2. Occurrence of the first seizure >1 week after TBI, and presentation with obvious clinical manifestations of epilepsy, such as disturbance of consciousness, hyperspasmia, and faintness
3. Definite findings on electroencephalogram (EEG)
4. Age between 20 and 70 years
5. Normal drug regimen and auricular electroacupuncture
6. Complete medical records

### 2.3. Exclusion Criteria for Late PTE

The exclusion criteria were as follows:

1. Deviation from the criteria described above
2. Other causes of secondary epilepsy, such as cerebrovascular disease
3. Status epilepticus
4. Pregnancy epilepsy
5. Other organic disorders, such as hypertension, heart diseases, hematologic diseases, AIDS, and mental disorders

### 2.4. Interventions

Eighty-nine patients received the following basic treatment: (1) maintaining an open airway, nutrition support, vital sign monitoring, and active symptomatic treatments, such as anti-infection, antipyretic, and antispasmodic treatments; (2) reducing intracranial pressure according to patients' specific conditions; and (3) rehabilitation training for eligible patients.

Patients who received edaravone (H20050280, Jiangsu Simcere Pharmaceutical Co., Ltd. China) treatment were administered edaravone 30 mg + 0.9% sodium chloride as a 100-ml intravenous infusion, twice a day, for 14 days for a course of treatment. Patients receiving sodium valproate (H43020874, Hunan Xiangzhong Pharmaceutical Co., Ltd., China) treatment were administered sodium valproate 100 mg orally, three times a day. The dose of sodium valproate was increased by 5 mg/d per week and gradually increased to a maximum dose of 200–400 mg, three times a day, during the hospitalization period.

Acupuncture was performed by licensed acupuncturists. The patients treated with auricular electroacupuncture included some who also received drug treatment, based on their specific conditions. The acupoints “Shenmen” and “Xin” were used, referring to the international standardization of auricular acupuncture points. Disposable stainless-steel needles (0.25 *∗* 25; Hwato, Suzhou New District, P. R. China) were inserted into the acupoints to a level of approximately 3 mm, parallel to the skin. The needles were connected to an electric acupuncture apparatus (Model SDZ-IIB; Hwato, Suzhou New District, P. R. China) with 1 Hz continuous wave current, to make the external ear tremble slightly. The needles were left in place for 30 minutes for each session. Electroacupuncture was performed once a day, on alternate ears, during the hospitalization period.

### 2.5. Outcome Measures

The main outcome in the study was the effect of auricular electroacupuncture on the incidence of late PTE. The secondary outcomes were the effects of sodium valproate and edaravone on auricular electroacupuncture in the treatment of late PTE. Efficacy was assessed according to the incidence of late PTE in the auricular acupuncture group and the control group. An obvious effect was defined as a significant reduction in the incidence and a reduction of >75% in the frequency of late PTE. Effectiveness was defined as a reduction in the incidence of late PTE and a reduction of 50–75% in its frequency. Ineffectiveness was defined as a lack of significant improvement or aggravation of symptoms.

### 2.6. Statistical Analyses

The raw data of each case were collated using Microsoft Excel (Office 2003). All statistical calculations were performed using SPSS v.17.0 (SPSS, Inc., Chicago, IL, USA). Correlations between the incidence of late PTE and gender, cause, and the use of auricular electroacupuncture, sodium valproate, and edaravone were evaluated by Pearson's correlation test. Multivariate logistic regression analysis was used to analyze the relationship between the positive results and the incidence of late PTE. The independent samples *t*-test was used for comparison of age between the auricular electroacupuncture group and the control group. Data are expressed as mean value ± standard deviation. The intergroup rate was tested by Pearson's correlation test. A *P* value <0.05 was considered statistically significant.

## 3. Results

### 3.1. Univariate Analysis

Based on the inclusion and exclusion criteria, 89 cases were included in the study. Seventy of the 89 cases with late PTE developed epilepsy; thus, the incidence rate was 88.76%. Univariate analysis was employed to analyze the relationship of age, cause of the injury, and the use of auricular electroacupuncture, sodium valproate, and edaravone with late PTE (Table 1). Only the use of auricular electroacupuncture, sodium valproate, and edaravone showed significant association with late PTE (*P* < 0.05).

### 3.2. Multivariate Logistic Regression Analysis

The three statistically significant factors identified in the univariate analysis were further investigated by multivariate logistic regression analysis (Table 2). The use of auricular electroacupuncture significantly reduced the incidence of late PTE (odds ratio [OR] = 50.886, 95% confidence interval [CI]: 7.808–331.607, *P* < 0.01).

### 3.3. Patient Characteristics

The 89 included patients were divided into the auricular electroacupuncture group and the control group, depending on whether auricular electroacupuncture was performed. The GCS was lower in the auricular electroacupuncture group than in the control group (*P* < 0.05), while other baseline characteristics were similar between these groups (*P* < 0.05; Table 3).

### 3.4. Effectiveness of Auricular Electroacupuncture

**Figure 2:**
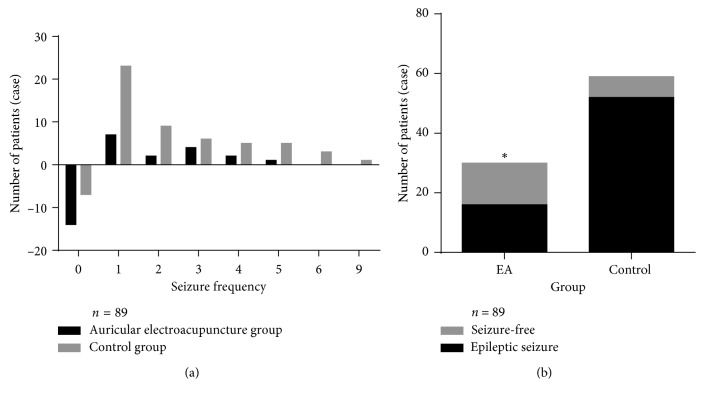
(a) The seizure frequency was concentrated on 0-1 in the auricular electroacupuncture group and 1-2 in the control group. (b) In contrast, the auricular electroacupuncture group had a significantly greater number of seizure-free cases (*P* < 0.01, *χ*^2^ test). Fourteen patients (46.67%) were seizure-free in the auricular electroacupuncture group, while 7 patients (11.86%) were seizure-free in the control group. ^*∗*^*P* < 0.01.

A *χ*^2^ test showed that the rate of effective treatment in the auricular electroacupuncture group was much higher than that in the control group (*P* < 0.05) (Table 4). There were more seizure-free cases in the auricular electroacupuncture group than in the control group (*χ*^2^ = 13.361, *P* < 0.01) (Figure 2).

### 3.5. Effect of Sodium Valproate on the Outcome of Auricular Electroacupuncture

**Figure 3:**
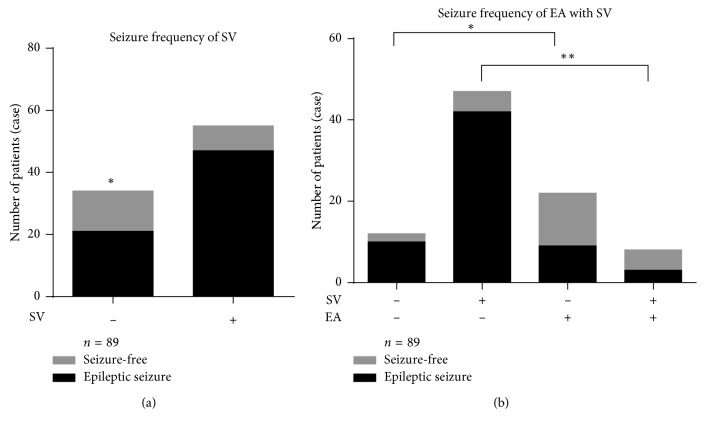
(a) In the 89 cases, significantly more seizure-free cases were observed among patients who did not receive sodium valproate (SV; *P* < 0.05, *χ*^2^ test). (b) The seizure-free rate was increased in patients who received auricular electroacupuncture (EA) and sodium valproate (*P* < 0.01, *χ*^2^ test). In the 30 cases who received EA, the seizure-free rate was not significantly reduced by sodium valproate, which was also seen in the 59 cases of the control (non-EA) group. The 55 cases treated with sodium valproate who received EA showed significantly increased seizure-free rates (*P* < 0.01, *χ*^2^ test). The 34 cases who did not receive sodium valproate showed a similar result (*P* < 0.05, *χ*^2^ test). ^*∗*^*P* < 0.05, ^*∗∗*^*P* < 0.01.

In the 89 cases overall, the seizure frequency was much lower in patients who did not receive sodium valproate (*χ*^2^ = 6.541, ^*∗*^*P* < 0.05) (Figure 3(a)). The seizure frequency in patients treated with auricular electroacupuncture was significantly lower than that in patients who were not treated with auricular electroacupuncture, regardless of whether sodium valproate was also used (*χ*^2^ = 4.078, ^*∗*^*P* < 0.05; *χ*^2^ = 14.859, ^*∗∗*^*P* < 0.01) (Figure 3(b)).

### 3.6. Effect of Edaravone on the Outcome of Auricular Electroacupuncture

**Figure 4:**
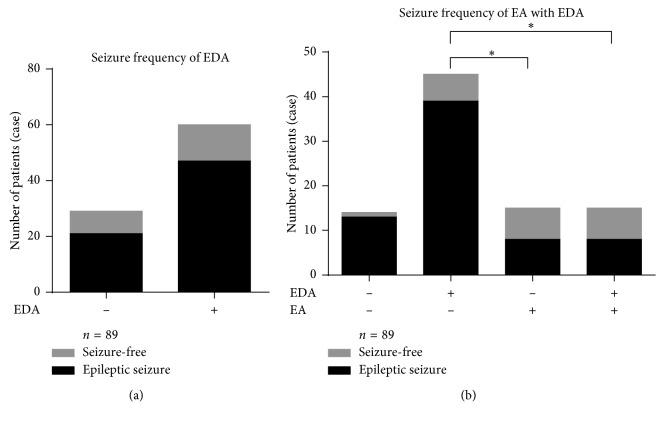
(a) Among the 89 cases, no difference was found between the cases treated with or without edaravone (EDA; *P* > 0.05, *χ*^2^ test). (b) The proportion of seizure-free cases was greater among patients who received auricular electroacupuncture (EA) and edaravone (*P* < 0.05, Fisher's exact test). In the 30 cases who received auricular electroacupuncture, the seizure-free cases did not increase significantly with the use of edaravone, as also seen in the 59 cases of the control (non-EA) group. Among the 60 patients who were treated with edaravone and received auricular electroacupuncture, significantly increased seizure-free cases were observed (*P* < 0.05, Fisher's exact test). Seizure frequency (53.33%) was lower in the auricular electroacupuncture group treated without edaravone than that (86.67%) in the group treated with edaravone only (*P* < 0.05, Fisher's exact test). ^*∗*^*P* < 0.05.

Compared with the cases who did not receive auricular electroacupuncture, the rates of the cases with auricular electroacupuncture were significantly reduced, regardless of the use of edaravone (*χ*^2^ = 5.532, ^*∗*^*P* < 0.05) (Figure 4(b)).

## 4. Discussion

We retrospectively analyzed the clinical efficacy of ABVN via auricular electroacupuncture for the treatment of PTE and evaluated the influence of sodium valproate and edaravone on the anti-PTE effects of this treatment. In this retrospective study of 89 cases, univariate analysis showed a significant correlation between the use of auricular electroacupuncture, sodium valproate, and edaravone and a reduced incidence of late PTE. Furthermore, among these three treatments, multivariate logistic regression analysis demonstrated that only the use of auricular electroacupuncture could reduce the incidence of late PTE (*P* < 0.01).

PTE is a serious complication after TBI and is aggravated by the severity of TBI. Central nervous system damage is associated with the evolution of late PTE [21]. Late PTE can have adverse effects on psychology, quality of life, and social activities, and can even lead to death [22]. Thus, it is important to take preventive and therapeutic measures against PTE. PTE can be triggered in the early stage of TBI, and thus AEDs are usually administered prophylactically after TBI. Classic AEDs are phenytoin, sodium valproate, and carbamazepine. In addition to AEDs, epilepsy resection surgery, neurosurgical radiotherapy, and chemotherapy are also used as treatment. In recent years, VNS has come to be recognized as another valuable technique for controlling seizures. Exploring the clinical efficacy of therapies is, therefore, clinically relevant for improving the prevention and treatment of late PTE.

Studies have shown that AEDs such as phenytoin and carbamazepine can only suppress the incidence of early PTE, but do not reduce the risk of late PTE, even during treatment [23, 24]. AEDs have some side effects, including psychiatric disorders, neurological disorders, gastrointestinal disorders, and skin and appendage disorders [25]. Nevertheless, due to the limitations of clinical treatment, AEDs remain the first choice for epilepsy treatment.

When we compared the basic information of the auricular electroacupuncture group and the control group, the auricular electroacupuncture group had a lower GCS score (*P* < 0.05). The severity of TBI is mainly classified by the GCS, but this scale is not particularly accurate [26]. In patients with severe TBI combined with multiple injuries, the GCS has low sensitivity, and the anatomical scoring system of the Abbreviated Injury Scale (AIS) is recommended [27]. The lower GCS in the auricular electroacupuncture group may be related to other factors. Age affects the GCS score in patients with TBI. In cases with similar TBI severity, elderly patients (≥65 years) had better GCS scores than younger patients (18–64 years), and GCS scores of the elderly patients were higher at each AIS level and were more apparent in cases with severe injury (AIS 5) [28, 29].

The present study showed that the total clinical effectiveness of auricular electroacupuncture for epilepsy treatment was significantly higher than that of treatment not including auricular electroacupuncture (*P* < 0.05). The proportion of seizure-free patients in the auricular electroacupuncture group was significantly greater than that in the control (non-electroacupuncture) group (*P* < 0.01). Our findings provide clinical evidence that ABVN via auricular electroacupuncture (continuous 1 Hz pulses for 30 minutes) significantly reduced the seizure frequency in late PTE. Furthermore, edaravone and AEDs such as sodium valproate were ineffective for suppressing the epileptiform activity of late PTE, which was consistent with previous studies that reported that sodium valproate had no positive effect against late seizures, while edaravone just plays a neuroprotective role, including the prevention of neurological deficits, apoptosis, and suppression of oxidative stress [23, 30]. Moreover, we also found that sodium valproate and edaravone had no influence on reduction of the incidence of late PTE, as compared to the effect of auricular electroacupuncture.

Vagus nerve stimulation targeting the left cervical vagus trunk has been approved by the FDA for treating intractable epilepsy [31]. Anatomical studies have demonstrated that the sensory vagus nerve projects to the NTS [32]. Currently, the vagus-NTS pathway is considered to be an essential target for treatment of epilepsy. However, VNS is applied to only about one-third of patients because stimulation of the cervical vagus trunk causes side effects, including hoarseness, cough, dyspnea, pain, paresthesia, nausea, and headache [33].

The ABVN is the only peripheral branch of the vagus nerve [14]. The NTS pathway is considered to be pivotal to the antiepileptic effect of auricular acupuncture [20]. Recently, an fMRI study provided evidence that the central projections of the ABVN are consistent with the “classical” central vagal projections and can be accessed noninvasively via the external ear in humans [34]. Neurophysiological studies have shown that parasympathetic efferent activities participate in antiepileptic effects [9]. As the ABVN carries parasympathetic nerve fibers mainly concentrated on the concha, the acupoints “Shenmen” and “Xin,” located on the external ear, were selected in this study. Some studies of transcutaneous vagus nerve stimulation by applying surface electrodes or acupuncture needles (ta-VNS) in patients have also demonstrated antiepileptic effects [18, 35]. During ta-VNS, sympathetic nerve outflow is reduced [36], which is critical for adjusting parasympathetic nerve activity.

Some recent studies suggested that inflammation possibly plays an important role in epilepsy [37]. When TBI occurs, the brain initiates an immediate neuronal and glial response, leading to an imbalance between excitatory and inhibitory neurotransmission, in which inflammation and increased blood–brain barrier permeability potentially play an important role [3]. The cholinergic anti-inflammatory pathway is involved in neural inhibition of inflammation via the efferent vagus nerve, by interacting with macrophage *α*7-nicotinic acetylcholine receptors (*α*7nAChR) [38]. The neural anti-inflammatory pathway, which includes the vagus nerve, central nervous system, and *α*7nAChR, is termed the inflammatory reflex [39]. This pathway communicates broadly with the neuroendocrine pathway, controlling systemic and local inflammation. The anti-inflammatory effects of auricular electroacupuncture can be involved in the mechanism underlying epileptic inhibition in late PTE.

Our study has some limitations. First, this was a retrospective analysis that was limited by small study size. The single hospital location made it difficult to obtain a large number of cases about late PTE following severe TBI. Second, depending to the clinical features of late PTE, patients received auricular electroacupuncture as a long-term regular treatment during the hospitalization period. Therefore, there were differences in the course of treatment for patients receiving auricular electroacupuncture. Since our study had a nonrandom, open trial design, the treatment plan for each patient depended entirely on the condition and the patient's wishes. Thus, some bias is likely in the results. Third, we have recognized that the auricular acupuncture had a multitarget therapeutic effect. Besides epilepsy, auricular acupuncture treatment could also regulate the visceral function, which has a greater impact on the patient's vital signs and biochemical indicators in the early stages of TBI. We only explored the late PTE in the late stage of severe TBI. The impact of auricular acupuncture on the visceral function in the late stage of TBI is worthy of further investigation.

Compared with sodium valproate, edaravone, and conventional VNS, auricular electroacupuncture is effective for reducing the incidence of late PTE via the stimulation of the ABVN. The use of auricular electroacupuncture can help to avoid the side effects of VNS, and it is a safe therapeutic option for PTE when performed by qualified doctors.

## Figures and Tables

**Table 1:** Univariate analysis of cases with and without seizures among 89 cases with late PTE.

Influencing factors	Cases (*n*)		*P* value
	Seizure	Seizure-free	
Total cases	70	19	
Gender			
Male	53	6	0.431
Female	17	3	
Cause			
Car accident	44	8	0.104
Non-car accident	26	11	
Auricular electroacupuncture			
With	12	18	≤0.001
Without	58	1	
Sodium valproate			
With	45	10	0.036
Without	21	13	
Edaravone			
With	52	9	0.025
Without	18	10	

**Table 2:** Multiple regression analysis of cases with seizure among 89 cases with late PTE.

Dependent variable	*β* value	S.E	OR value	OR 95% CI	*P* value
With auricular electroacupuncture	3.930	0.956	50.886	7.809–331.607	≤0.001
With sodium valproate	−0.436	0.832	0.647	0.127–3.299	0.600
With edaravone	0.364	0.702	1.425	0.360–5.640	0.614

**Table 3:** Baseline characteristics of the study patients.

Characteristic	No. (%) of patients	
	Auricular electroacupuncture group (*n* = 30)	Control group (*n* = 59)
Gender		
Female	5 (16.67)	15 (25.42)
Male	25 (83.33)	44 (74.58)
Age (years), mean ± SD	43.37 ± 13.69	46.63 ± 11.73
Cause of disease		
Accident	18 (60.00)	40 (67.80)
Fall	5 (16.67)	11 (18.64)
Others	7 (23.33)	8 (13.56)
Injury types		
Brain contusion with intracranial hematoma	9 (30.00)	17 (28.81)
Brain contusion with subdural hematoma	8 (26.67)	18 (30.51)
Brain contusion bead retinal hemorrhage	6 (20.00)	10 (16.95)
Diffuse axonal injury	3 (10.00)	6 (10.17)
Glasgow Coma Scale		
3–5	25 (83.33)^a^	23 (38.98)
6–8	5 (16.67)^a^	36 (61.02)
Injury location		
Frontal lobe	3 (10.00)	9 (15.25)
Temporal lobe	8 (26.67)	17 (28.81)
Frontotemporal lobe	14 (46.67)	26 (44.07)
Occipital lobe	5 (16.67)	7 (11.86)
Surgery		
Yes	14 (46.67)	33 (55.93)
No	15 (50.00)	26 (44.07)

Notes: ^a^*P* < 0.05, significant difference between the groups.

**Table 4:** Comparison of curative effects between the two study groups (*n*).

Group	*n*	Obvious effect	Effectiveness	Ineffectiveness	Total effective rate
Auricular electroacupuncture	30	21	6	3	27 (90.00)^a^
Control	59	30	15	14	45 (76.27)

Notes: The curative effects in the auricular electroacupuncture group were significantly higher than those in the control group (*P* < 0.01, *χ*^2^ test). Thirty patients received auricular electroacupuncture at “Shenmen” and “Xin” points, by connecting to an electric acupuncture apparatus with 1 Hz current to make the external ear tremble slightly. The needles were left in place for 30 minutes for each session. The method was performed once a day, alternating between both ears. All 89 patients received base treatment. The treatment efficiency of electroacupuncture in the two groups was 90.00% and 76.27%; ^a^*P* < 0.05.

## Data Availability

The data used to support the findings of this study are included within the article.
